# Inhibitory effects of flavonoids isolated from *Sophora flavescens* on indoleamine 2,3-dioxygenase 1 activity

**DOI:** 10.1080/14756366.2019.1640218

**Published:** 2019-08-18

**Authors:** Mincheol Kwon, Sung-Kyun Ko, Mina Jang, Gun-Hee Kim, In-Ja Ryoo, Sangkeun Son, Hyung Won Ryu, Sei-Ryang Oh, Won-Kyu Lee, Bo Yeon Kim, Jae-Hyuk Jang, Jong Seog Ahn

**Affiliations:** a Anticancer Agent Research Center, Korea Research Institute of Bioscience and Biotechnology, Cheongju, Korea;; b Department of Biomolecular Science, KRIBB School of Bioscience, Korea University of Science and Technology, Daejeon, Korea;; c Natural Medicine Research Center, Korea Research Institute of Bioscience and Biotechnology, Cheongju, Korea;; d New Drug Development Center, Osong Medical Innovation Foundation, Cheongju, Korea

**Keywords:** Indoleamine 2,3-dioxygenase 1 (IDO1), IDO1 inhibitor, *Sophora flavescens*, flavonoid

## Abstract

Indoleamine 2,3-dioxygenase 1 (IDO1), a tryptophan catabolising enzyme, is known as a tumour cell survival factor that causes immune escape in several types of cancer. Flavonoids of *Sophora flavescens* have a variety of biological benefits for humans; however, cancer immunotherapy effect has not been fully investigated. The flavonoids (1–6) isolated from *S. flavescens* showed IDO1 inhibitory activities (IC_50_ 4.3**–**31.4 µM). The representative flavonoids (**4–6**) of *S. flavescens* were determined to be non-competitive inhibitors of IDO1 by kinetic analyses. Their binding affinity to IDO1 was confirmed using thermal stability and surface plasmon resonance (SPR) assays. The molecular docking analysis and mutagenesis assay revealed the structural details of the interactions between the flavonoids (1–6) and IDO1. These results suggest that the flavonoids (1–6) of *S. flavescens*, especially kushenol E (**6**), as IDO1 inhibitors might be useful in the development of immunotherapeutic agents against cancers.

## Graphical Abstract

**Figure:**
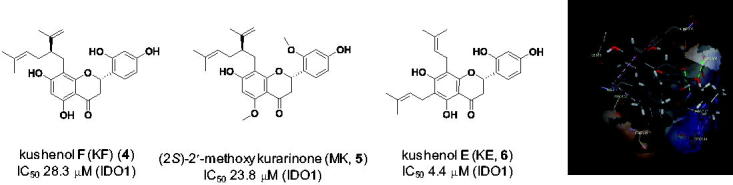

## Introduction

*Sophora flavescens* has been used traditionally to treat skin diseases, viral hepatitis, and cancer owing to the presence of medicinal components such as alkaloids and flavonoids^1–3^. Although many studies have focused on the medicinal properties of alkaloids than those of flavonoids, some studies have evaluated flavonoids and their bioactive mechanisms of action. The polyphenol structure of flavonoids is responsible for their various pharmacological activities, which are elicited by chelating metal ions or scavenging free radicals^4–8^. Indeed, it has been proved that the existence of prenyl and lavandulyl residue could intensify the pharmacological activity of the flavonoids^9^. Because prenylation and lavandulylation increase lipophilicity, which results in an increased membrane-binding affinity and a stronger interaction with the target proteins^10^^,^^11^.

The _L_-Tryptophan (_L_-Trp) is an essential amino acid and can be converted to kynurenine (Kyn), a natural ligand of the aryl hydrocarbon receptor, by Trp-catabolising enzymes such as indoleamine 2,3-dioxygenase 1 (IDO1), indoleamine 2,3-dioxygenase 2 (IDO2), and tryptophan 2,3-dioxygenase (TDO)^12^. IDO1 is a heme-containing enzyme and is detected in various organs such as spleen, liver, small intestine, kidney, lung, brain, thymus and colon^13^^,^^14^. However, the expression of IDO1 in cancers is rapidly increased by inflammatory cytokines such as interferons (IFNs) and tumor necrosis factors (TNFs)^15^^,^^16^. IDO1 contributes to immune-regulation by converting _L_-Trp to Kyn and inducing amino acid starvation in cancer environment. In addition, IDO1 induces differentiation of regulatory T cells and avoids the immune responses^16–18^.

In fact, some flavonoids of *S. flavescens* have been reported as tyrosinase inhibitors, anti-inflammatory and anti-oxidant substances, but have not been reported as IDO1 inhibitors yet^19^^,^^20^. In this study, we demonstrate the identification of flavonoids (**1–6**) from *S. flavescens* as a novel class of IDO inhibitor.

## Material and methods

### Flavonoids of *S. flavescens*

The flavonoids of *S. flavescens* were purified by the method of Lee et al.^21^. The isolated flavonoids (**1**–**6**) were identified from spectroscopic data and chemical evidence through comparisons with previous reports^21^^,^^22^.

### Purification of IDO1

The *Escherihia coli* DH5α and T7 strain were used for gene cloning and protein production, respectively. The human IDO1 clone was provided from Korea Human Gene Bank, Medical Genomics Research Center, KRIBB, Korea. The full-length of IDO1 gene was amplified by PCR using pfu DNA polymerase (Thermo Fisher, , Waltham, MA). The PCR product from IDO1 mRNA was cloned into expression vector pET28a to obtain pET28a-IDO1 and confirmed using DNA sequencing (Cosmogenetech, Seoul, Korea). The *E. coli* T7 competent cells were transformed with this plasmid and expression of IDO1 protein was induced with 0.2 mM isopropyl-β-D-1-thiogalactopyranoside (IPTG, Sigma, St. Louis, MO) for 4 h at 37 °C. The expressed IDO1 protein was purified using Ni-NTA agarose (Qiagen, Valencia, CA) at 4 °C. The eluted IDO1 protein was dialysed using storage buffer (20 mM Tris-HCl buffer (pH 8.0 and 200 mM NaCl)). The purified IDO1 protein was analysed with SDS-PAGE.

### IDO1 enzyme assay

The enzyme assays were performed with previous descripted in Takikawa et al.^23^. Briefly, 200 µL of the reaction mixture contained potassium phosphate buffer (50 mM, pH 6.5), ascorbic acid (20 mM), methylene blue (10 µM), catalase (10 mM), purified recombinant human IDO1 enzyme (5 µg/mL) and _L_-Trp (200 µM). The metabolites were serially diluted in dimethyl sulfoxide (DMSO, 0.5%, v/v) and transferred to a 96-well plates. The reaction was conducted at 37 °C for 1 h and stopped by adding 40 µL of trichloroacetic acid (30%); the solution was then heated at 65 °C for 15 min. The plates were centrifuged at 4000 rpm for 15 min to remove debris. Next, the supernatant (125 µL) from each well was transferred to a 96-well plates. *p*-dimethylaminobenzaldehyde (125 µL; 2%, v/v) in acetic acid was then added to each well, and the absorbance value of the plate was read at a wavelength of 480 nm using a SpectraMAX-190 ELISA reader (Molecular Devices, Sunnyvale, CA).

### Cell culture

HeLa (Human cervical cancer cells) cells were purchased from the American Type Culture Collection (ATCC) and cultured in DMEM supplemented with fetal bovine serum (FBS, 10%) and penicillin–streptomycin (1%). The cells were maintained at 37 °C with 5% CO_2_.

### Cell-based IDO1 assay

HeLa cells were seeded into 48-well plates (2 × 10^4^ cells/well). Next, the cells were stimulated with human recombinant IFN-γ (50 ng/mL, R&D Systems,  Minneapolis, MN) in complete DMEM for 24 h. After replacing the medium with DMEM containing IFN-γ, the cells were treated with the flavonoids (**1–6**) or DMSO (0.5%, v/v) for 6 h, and then the supernatant (125 µL) from each well was transferred to a 96-well plate. The cell-based assay was similarly performed to the IDO1 enzyme assay.

### Cell viability test

The cell viability was tested using the EZ-Cytox (Daeil Lab, Seoul, Korea) reagent according to the manufacturer’s recommendations. Briefly, HeLa cells (5 × 10^3^ cells/well) were cultured in 96-well plates and pretreated with human recombinant IFN-γ for 24 h. After following an overnight incubation, each of the cells was treated with serially diluted flavonoids (**4–6**) for 6 h. EZ-Cytox (10 µL) was added to each of the wells of the plate and incubated for 1 h. The absorbance was determined at 450 nm with SpectraMAX-190 ELISA reader (Molecular Devices, Sunnyvale, CA). The cell viability was normalised to the control group.

### IDO1 enzyme kinetics assay

The kinetics assay was performed by adding purified protein to the reaction mixture (200 µL) which contained a final concentration of potassium phosphate buffer (50 mM, pH 6.5), ascorbic acid (20 mM), catalase (10 mM), methylene blue (10 µM), and distilled water up to 200 µL. Various concentrations of _L_-Trp as substrate of IDO1 were added to the reaction mixture with four different concentrations of flavonoids (0–45 µM). After incubation at 37 °C for 1 h, the dissociation constant (*κ_i_*) of the three flavonoids were determined by measuring the activity at five concentrations of _L_-Trp (0 to 50 µM).

### Thermal shift assay

The thermal stability of proteins was measured by SYPRO-Orange (Thermo Fisher Scientific, Waltham, MA) as previously described in Soderhole et al.^24^. Briefly, IDO 1 enzyme (5 µg/mL) was mixed with 30 µM of kushenol F (KF, **4**), (2*S*)-2'-methoxy kurarinone (MK, **5**), and kushenol E (KE, **6**), or serially diluted KE (**6**) in 20 µL reaction buffer containing potassium phosphate buffer (50 mM) and sodium chloride (100 mM), and then incubated for 1 h. The measurement of protein denaturation via an increase in temperature was determined using real-time PCR device (CFX96 Real Time System, BioRad, Hercules, CA) using the FRET channel. In the presence of IDO1 enzyme, SYPRO-Orange has a maximum excitation near 490 nm and a maximum emission near 600 nm. The heating rate and fluorescence reading were 0.5 °C/10 s depending on the temperature range 25–95 °C.

### Surface plasmon resonance (SPR) assay

The SPR assay was performed with 10 mM stock solution and five serially diluted concentrations of KE (**6**) in PBS and 5% DMSO at 25 °C using a Reichert instrument (SR7500DC System, USA) using a sensor chip CM5 (GE Healthcare, Chicago, IL) (Woojung BSC Inc, Seoul, Korea). Amine coupling of His-IDO1 (WT and P314A) was performed with 0.2 M 1-ethyl-3-(3-dimethylaminopropyl)-carbodiimide, 0.05 M N-hydroxysuccinimide, and 1 M ethanolamine (ph 8.5, GE Healthcare, Chicago, IL). The results were analysed using Scrubber software (version 2.0, BioLogic Software, Mundelein, IL).

### Molecular modelling

The crystal structure of IDO1 from the Protein Data Bank (PDB code 6AZU (holoenzyme form)) was used for docking simulations^25^. The structure of flavonoids (**1–6**) were built using the Maestro build panel and minimised using the Impact module of Maestro in the Schrödinger suite program^26^. The starting coordinates of IDO1 were further modified for docking. Energy-minimised flavonoids (**1–6**) were docked into the prepared receptor grid by LibDock in Discovery Studio 2018 suite (Biovia, San Diego, CA)^26^. Additionally, the software was used to create molecular graphics for inhibitor binding pocket and a refined docking model for KE (**6**).

### Site-directed mutagenesis

Point mutations were introduced using the Muta-Direct^TM^ mutagenesis kit (iNtRON, Seuol, Korea). Oligonucleotides for generation of Pro182 (P182A), Phe185 (F185A), double mutation (P182A and F185A), and Pro 314 (P314A) to alanine mutants (mutated residues are underlined) were as follows:

For P182A: 5′-CAATCAAAGTAATTGCTACTGTATTCAAGG-3′, 5′-CCTTGAATACAGTAGCAATTACTTTGATTG-3′.
For F185A: 5′-AATTCCTACTGTAGCCAAGGCAATGCAAAT-3′, 5′-ATTTGCATTGCCTTGGCTACAGTAGGAATT-3′.
For double mutation (P182A and F185A): 5′-AATCAAAGTAATTGCTACTGTAGCCAAGGCAATGCAAATG-3′, 5′-CATTTGCATTGCCTTGGCTACAGTAGCAATTACTTTGATT-3′.
For P314A: 5′- CATTAGAGTCAAATGCCTCAGTCCGTGAGT-3′, 5′- ACTCACGGACTGAGGCATTTGACTCTAATG-3′.

All construct sequences were confirmed before use.

## Results

### IDO1 inhibitory activities of the flavonoids from *S. flavescens*

**Figure 1.**
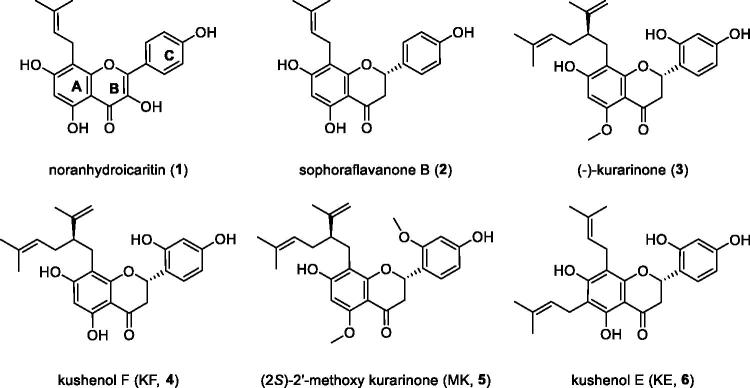
Chemical structures of the flavonoids (**1–6**) from *S. flavescens*.

The diverse therapeutic effects of flavonoids from *S. flavescens* indicated the possibility for cancer treatments and anti-inflammatory agents^19^^,^^20^^,^^27^. However, the IDO1 inhibitory activity has not yet been determined in *S. flavescens*. For this reason, all of the flavonoids (**1–6**) isolated from *S. flavescens* were tested for IDO1 inhibitory activity (Figure 1). The noranhydroicaritin (**1**), sophoraflavanone B (**2**), (−)-kurarinone (**3**), kushenol F (KF, **4**), (2*S*)-2'-methoxy kurarinone (MK, **5**), and kushenol E (KE, **6**) from *S. flavescens* exhibited IDO1 inhibitory activities (Table 1). Our results show some interesting fact of the structure–activity relationship. The prenyl group on A ring in the flavonoids appears to play pivotal roles in the inhibition of IDO1. This can be seen by comparing bi-prenylated flavonoid KE with lavandulylated flavonoids analogue (−)-kurarinone, KF and MK. The KE (IC_50_ = 7.7 µM) is three times more effective than (−)-kurarinone (**3**, IC_50_ = 23.4 µM), KF (**4**, IC_50_ = 25.4 µM) and MK (**5**, IC_50_ = 31.4 µM), respectively. The presence of a hydroxyl group at B and C ring is also important for inhibitory potency: i.e. compare (−)-kurarinone (**3**) versus MK (**4**, IC_50_ = 23.4 versus 31.4 µM) and sophoraflavanone B versus noranhydroicaritin (IC_50_ = 19.3 versus 23.5 µM). We also tested 1-methyl-tryptophan (1-MT) as a positive control and steppogenin, a non-prenyl and non-lavandulyl flavonoid backbone, as negative control.

**Table 1.** The inhibitory effects (IC_50_) of flavonoids from *S. flavescens* against recombinant human IDO1.

Compounds	His-hIDO1 IC_50_ (µM)
Noranhydroicaritin (**1**)	23.5 ± 2.2
Sophoraflavanone B (**2**)	19.3 ± 1.1
(−)-Kurarinone (**3**)	23.4 ± 1.3
Kushenol F (KF, **4**)	25.4 ± 4.4
(2*S*)-2′-Methoxy kurarinone (MK, **5**)	31.4 ± 1.3
Kushenol E (KE, **6**)	7.7 ± 6.2
1-Methyl-tryptophan (1-MT)^a^	423.3 ± 31.1
Steppogenin^b^	NA

^a^Positive control^28^.

^b^Steppogenin is a non-prenyl and non-lavandulyl backbone of flavonoids. Experiments were performed in triplicate and repeated three times with similar results (mean ± SD).

NA: no activity detected.

### Cell-based IDO1 inhibitory activity

**Figure 2.**
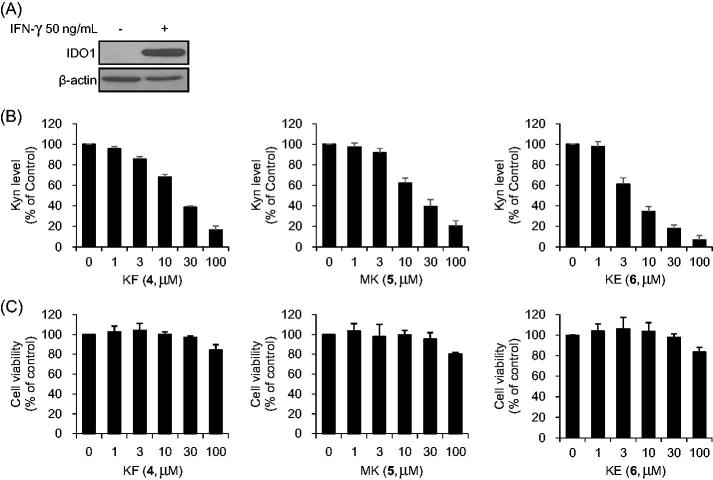
IDO1 inhibitory activity and cytotoxicity of flavonoids (**4–6**) were measured in HeLa cells. (A) IDO1 expression was confirmed in IFN-γ-treated HeLa cells. (B) Kynurenine (Kyn) production level was measured in IFN-γ-treated HeLa cells treated with various concentrations of KF, MK, and KE for 6 h. (C) Cell viability of IFN-γ-treated HeLa cells was measured by EZ-Cytox after treating with indicated concentrations of KF, MK, and KE for 6 h. Data are representative of three independent experiments. The experiments were performed in triplicate and repeated three times with similar results (mean ± SD).

To investigate IDO1 activity in tumour cell, HeLa cells were exposed to recombinant IFN-γ (50 ng/ml) to induce endogenous IDO1 expression and analysed the Kyn production level from _L_-Trp degradation by IDO1 (Figure 2(A)). The cell-based IDO1 enzyme inhibitory activities of flavonoids (1–6) were evaluated and a known IDO1 inhibitor, 1-MT was used as the positive control (Table 2). Three flavonoids, KF (**4**), MK (**5**), and KE (**6**) are more potent than other flavonoids (1–3) in cells (Table 2) and reduced Kyn levels in a dose-dependent manner; the corresponding IC_50_ values for inhibition were 28.3 ± 0.3, 23.8 ± 1.6 and 4.3 ± 0.3 µM, respectively (Figure 2(B)). As the flavonoids (4–6) were cytotoxic at prolonged exposure times, the cell-based IDO1 assay was performed for 6 h in order to reduce cytotoxicity, after treating with IFN-γ for 24 h (Figure 2(C)). KE (**6**) showed the strongest inhibitory effect on Kyn production (IC_50_ = 4.3 ± 0.3 µM).

**Table 2.** The inhibitory effects (IC_50_) of flavonoids from *S. flavescens* against cell-based IDO1 in IFN-γ-treated HeLa cells.

Compounds	His-hIDO1 IC_50_ (µM)
Noranhydroicaritin (**1**)	42.2 ± 3.5
Sophoraflavanone B (**2**)	83.6 ± 10.8
(−)-Kurarinone (**3**)	74.9 ± 4.9
Kushenol F (KF, **4**)	28.3 ± 0.3
(2*S*)-2′-Methoxy kurarinone (MK, **5**)	23.8 ± 1.6
Kushenol E (KE, **6**)	4.3 ± 0.3
1-Methyl-tryptophan (1-MT)^a^	1080.3 ± 210.3

^a^Positive control^28^. Experiments were performed in triplicate and repeated three times with similar results (mean ± SD).

### Inhibition kinetics

**Figure 3.**
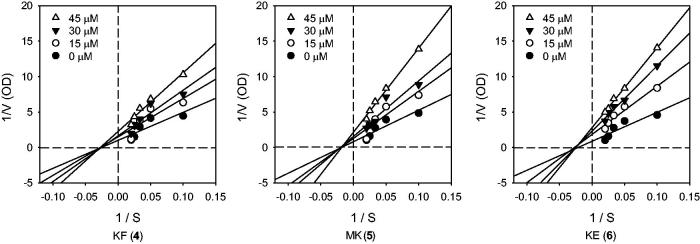
IDO inhibition kinetics of flavonoids (**4–6**). IDO1 enzyme was treated with 0 to 45 µM of each compound using 0–50 µM of _L_-Trp as the substrate. Experiments were performed in triplicate and repeated three times with similar results.

To investigate the inhibition mode, the kinetics of representative IDO1 inhibitory active flavonoids (**4**–**6**) were quantitatively analysed by the Lineweaver–Burk plots. The experiments were performed using _L_-Trp, as the substrate at a concentration of 0–50 µM and with the flavonoids at a concentration of 0–45 µM. This analysis (Figure 3) showed that *V*_max_ decreased without changing *K*_m_ in the presence of increasing concentrations of the inhibitors, as shown in the graph. The *x*-intercept, −1/*K*_m_, was unaffected by the inhibitor concentration, whereas 1/*V*_max_ became more positive. This behaviour indicates that flavonoids exhibit non-competitive inhibition characteristics for IDO1. The *κ_m_* value obtained in the kinetics assay was 21.7 ± 0.3 µM. The *κ_i_* values of these flavonoids were calculated using the quadratic equation of the Dixon plots (data not shown). The *κ_i_* values of KF (**4**), MK (**5**), and KE (**6**) were 20.2 ± 3.1, 13.7 ± 0.03, and 9.5 ± 0.6 µM, respectively (Figure 3). The flavonoids (**4**–**6**) showed a typical tendency of non-competitive inhibition behaviour.

### IDO1 binding assay of the flavonoids

**Figure 4.**
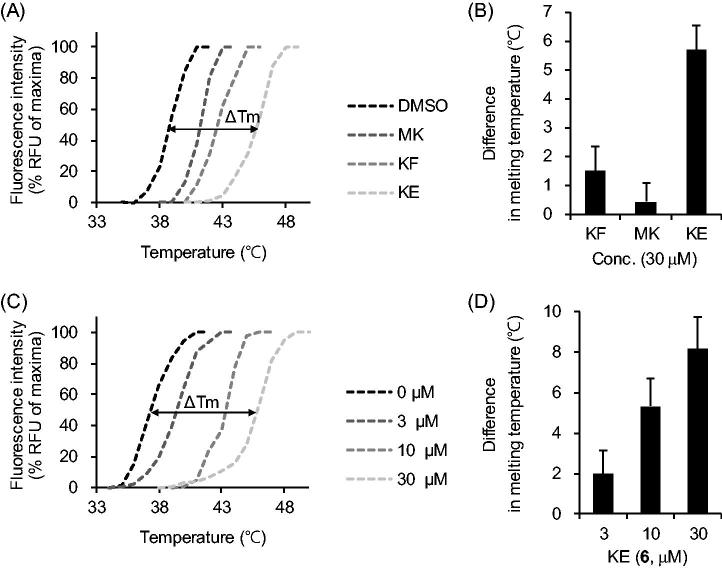
The flavonoids (**4–6**) directly bind to IDO1. (A and B) The flavonoids (**4–6**) were treated with SYPRO-Orange (10 µM) to purified recombinant his-IDO1. The fluorescence intensity of the binding was measured at increasing temperature (A) and the mean Δ*T*_m_ was calculated (B). (C and D) The fluorescence intensity of 3–30 µM KE was measured (C) and its mean Δ*T*_m_ was calculated (D). Experiments were performed in triplicate and repeated three times with similar results (mean ± SD).

The thermal stability assay has been widely used to confirm the interactions between compounds and their target proteins. The interaction of compound is expected to enhance the thermal stability of the protein^29^^,^^30^. The binding of representative flavonoids (**4**–**6**) to IDO1 was determined by the temperature stability analysis using SYPRO-Orange. The SYPRO-Orange binds to the hydrophobic residues in the unfolded regions of proteins, and is denatured by temperature^30^. Then we exposed the flavonoids (**4**–**6**) to IDO1 with SYPRO-Orange in order to confirm whether they change unfolding temperature of IDO1 enzyme without _L_-Trp. The flavonoids (**4**–**6**) were increased the mean melting temperature compared to the DMSO-treated group by +1.5 ± 0.9, +0.4 ± 0.7, and +5.7 ± 0.8 °C, respectively (Figure 4(A,B)). Since KE (**6**) exhibited the strongest IDO1 inhibitory activity, we tested experiments with various concentrations of KE (Figure 4(C,D)). These binding results indicate that the flavonoids (**4**–**6**), especially KE (**6**) increase the structural stability of the IDO1 enzyme by directly binding.

### Molecular docking analysis

**Figure 5.**
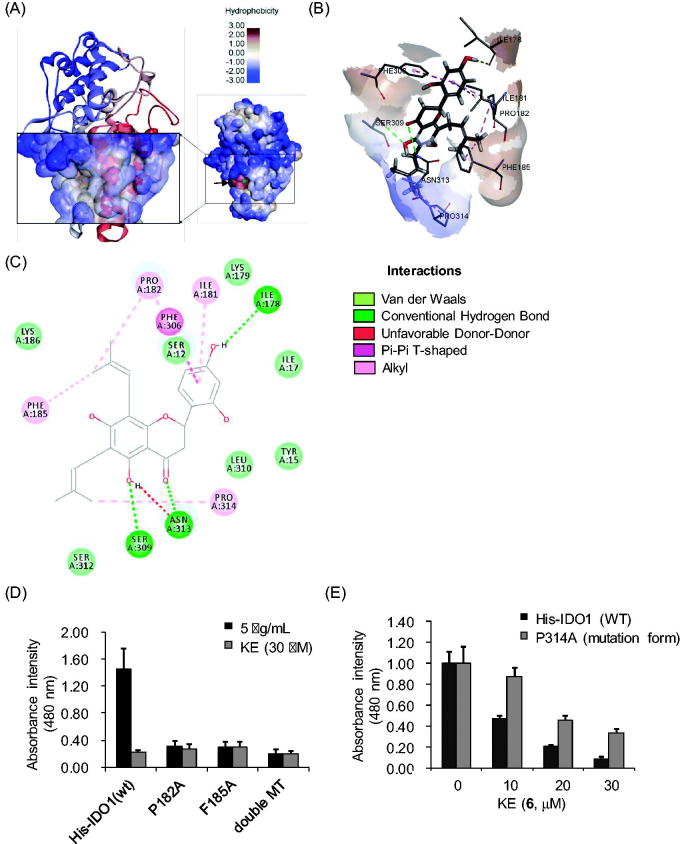
Illustration of intermolecular interaction between lowest binding energy conformations of KE in the allosteric site of the IDO1 enzyme (PDB code: 6AZU). (A) IDO1 surface structure represented by hydrophobicity. Right small IDO1 structure of red area (arrow) is selected allosteric site for docking analysis. The brown surface area of the left IDO1 structure shows that this allosteric site is a hydrophobic region compared to the surrounding area. (B) Interaction surface structure in stereo view (3D). KE shown in black sticks. Dotted lines are non-covalent interaction of KE with IDO1. (C) 2D diagram of KE with IDO1 interaction. (D) The IDO1 (wild-type) and three forms of mutation were tested with or without KE (30 µM). (E) The IDO1 (wild-type) or P314A mutant forms were tested with various concentrations (10, 20, and 30 µM) of KE. The experiments were performed in triplicate and repeated three times with similar results (mean ± SD).

To visualise the binding of flavonoids (**1–6**) to the IDO1, enzymes were applied to the molecular docking analysis. The molecular docking analysis using LibDock program revealed the mode of binding of flavonoids (**1–6**) at the allosteric site of IDO1 (Figure 5(A,B)). The surface expressed in brown represents the hydrophobic region and the region blue represents the hydrophilic region. The flavonoids (**1–6**) were calculated to be docked in the hydrophobic valley region (arrow) of the enzyme. For this docking analysis, the ligand binding sites in IDO1 were searched and there were five candidate sites (data not shown). Among them, however, we selected the sites where six kinds of flavonoids were commonly combined and where a site had the highest stable energy. The Ser^309^, Asn^313^, and Ile^178^ residues in the binding pocket played critical roles in the high-affinity binding of the flavonoids (**1–6**) through hydrogen-bond formation. Furthermore, pi–pi stacking interactions were observed between Phe^306^ and the flavonoids (**1**–**6**). However, we anticipated that Pro^182^, Phe^185^, and Pro^314^ were more important for binding of the flavonoids (**1**–**6**). This was because prenyl and lavanduyl residues of the flavonoids interacted with the site of the IDO1 enzyme. In Figure 5(C), the top prenyl residue of KE (**6**) was shown to bind to Pro^182^ and Phe^185^, and the bottom prenyl residue was shown to bind to Pro^314^ through alkyl formation with A ring on the prenyl and lavanduyl groups, but other flavonoids (**1**–**5**) interacted only with Pro^182^ and Phe^185^ (Figure 5(C)). These docking results suggest that the flavonoids (**1**–**6**), especially KE (**6**), have the most inhibitory activity against IDO1.

### Mutagenesis assay

To confirm which binding sites were crucial for IDO1 inhibition by these flavonoids, we performed mutagenesis in the His-IDO1 structure. According to the molecular docking results, Pro^182^, Phe^185^, and Pro^314^ residues were identified as the binding sites for the KE (**6**) bi-prenyl residues. We generated four types of mutations, P182A, F185A, P182A/F185A double mutation, and P314A. Next, we confirmed that Pro^182^ and Phe^185^ were the crucial binding sites of the flavonoids, because their mutagenesis (P182A, F185A, and P182A/F185A) eliminated the IDO1 activity (Figure 5(D)). Indeed, the mutant form of His-IDO1 (P314A) increased IC_50_ value of KE to 29.8 ± 1.8 µM (Figure 5(E)). These results indicated that Pro^182^ and Phe^185^ sites were highly important in the IDO1 structure, and Pro^314^ increased the stability of KE (**6**) binding to IDO1.

### SPR analysis of KE to IDO1 interaction

**Figure 6.**
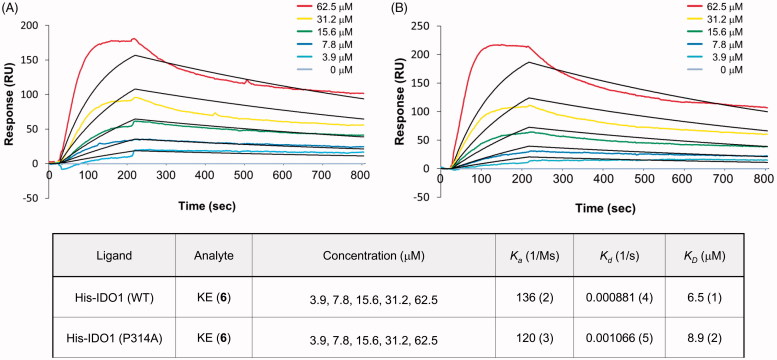
Interaction of IDO1 with KE was determined using the surface plasmon resonance (SPR) assay. (A and B) The SPR assay of binding of KE to His-IDO1 (WT) (A) and the mutant form of His-IDO1 (P314A) (B). KE at various concentrations was injected over the sensor chip surface immobilized with the His-IDO1 enzymes (WT and P314A mutant form). The KD value was 6.5 ± 4 (WT) and 8.9 ± 1 µM (P314 mutation form). The coloured lines represent original data curves and black lines represent fitted data curves. Numbers in parentheses indicate standard errors.

To determine the interaction between IDO1 and KE (**6**), we utilised a surface plasmon resonance (SPR) assay. Specifically, His-IDO1 was linked to the sensor chip and then incubated with successive injections of the KE (**6**) solution at a flow rate of 30 µL/min in PBS buffer-containing 5% DMSO. The SPR results showed that KE (**6**) was strongly bound to WT IDO1 with a *K_D_* value of 6.5 ± 0.1 µM (Figure 6(A)), but more slightly bound to the mutant IDO1 P314A with a *K_D_* of 8.9 ± 0.2 µM (Figure 6(B)). The equilibrium dissociation constant (*K_D_*) was calculated from the ratio of the dissociation rate constant divided by the association rate constant (*K_D_* = *k_d_*/*k_a_*). Based on this, we confirmed the direct binding of KE (**6**) to IDO1.

## Discussion

In this study, the flavonoids (**1**–**6**) from *S. flavescens* showed inhibitory effects on IDO1, in both enzyme-based and cell-based assays. The representative flavonoids (**4–6**) were identified as non-competitive IDO1 inhibitors through kinetic analyses, and the direct interactions with IDO1 were confirmed by thermal stability assay and SPR assay. We checked the amount of active His-IDO1 through by measuring absorbance at 280/404 nm absorbance (data not shown), because IDO1 can exist in equilibrium in its forms of haeme-bound (holo-IDO1) and haeme-free (apo-IDO1) form. These results indicate that the half of the measured His-IDO1 amount was in fact the apo-form. However, this did not affect the results of the IDO1 enzyme kinetics assay, but could explain the high *K_D_* values obtained in the SPR assay.

Initially, we determined that the residues of the flavonoids (**1**–**6**) are important to IDO1 inhibitory activity, because steppogenin, a non-prenyl and non-lavandulyl flavonoid, does not affect IDO1 inhibitory activity. Molecular docking analysis supported the results of IDO1 enzyme assay. In the molecular docking results, the prenyl residues of active flavonoids have binding affinity to Pro^182^ and Phe^185^. The flavonoids (**1**–**6**) were found to be embedded in the inner side of IDO1 completely (Figure 5(A,B)). The interactions between IDO1 and KE (**6**) were accomplished by hydrogen bonds and alkyl interactions (Figure 5(C)). Most of the hydrogen bonds interacted with the flavonoid-backbone, but prenyl residues of the flavonoids (**1**–**6**) were bound to the Pro^182^ and Phe^185^ residues through alkyl interactions. In particular, Pro^314^ of KE (**6**) imparted a binding site on another prenyl residue (Figure 5(D,E)). The P314A mutant assay revealed why KE (**6**) had the strongest activity as compared to other flavonoids of *S. flavescens.* Therefore, flavonoid prenyl or lavandulyl residues exhibited IDO1 inhibitory activity, as a difference to steppogenin. Thus, we considered that the alkyl interactions by prenyl were important in IDO1 inhibition.

According to previous reports, substrate _L_-Trp binds to Arg^231^, Ala^264^, and Thr^379^ residues in IDO1; meanwhile, Ala^264^ and His^346^ residues are related with haeme-binding ability of IDO1^31^. As described above, the binding site of the flavonoids was considerably distant from the active site of IDO1, and, thus, the flavonoids could act as an allosteric site of IDO1 inhibition activity. In conclusion, this study demonstrated that the flavonoids of *S. flavescens* had inhibitory activity against IDO1 enzyme, especially KE (**6**). Therefore, these molecules should be further investigated due to their IDO1 inhibitor ability and their potential use in cancer immunotherapy.
